# Specific DNA methylation markers in the diagnosis and prognosis of esophageal cancer

**DOI:** 10.18632/aging.102569

**Published:** 2019-12-13

**Authors:** DaPeng Li, Lei Zhang, YuPeng Liu, HongRu Sun, Justina Ucheojor Onwuka, ZhiGang Zhao, WenJing Tian, Jing Xu, YaShuang Zhao, HongYu Xu

**Affiliations:** 1 Department of Epidemiology, Public Health School of Harbin Medical University, Harbin 150081, China; 2 Department of Otorhinolaryngology Head and Neck Surgery, The First Affiliated Hospital of Harbin Medical University, Harbin 150001, China; 3 Department of Gastroenterology, The First Affiliated Hospital of Harbin Medical University, Harbin 150001, China

**Keywords:** DNA methylation, biomarker, Barrett’s esophagus, esophageal cancer, LASSO

## Abstract

The early diagnosis and accurate prognosis prediction of esophageal cancer is an essential part of improving survival. However, these diseases lack effective and specific markers. A total of 1,744 samples of HumanMethylation450 data were integrated to identify and validate specific methylation markers for esophageal adenocarcinoma (EAC) and esophageal squamous cell carcinoma (ESCC) as well as for Barrett’s esophagus (BE) using The Cancer Genome Atlas and the Gene Expression Omnibus. The diagnostic and prognostic methylation classifiers were constructed by moderated t-statistics and the least absolute shrinkage and selection operator method. The diagnostic methylation classifier using 12 CpG sites was constructed in training set (377 samples) that could effectively discriminate samples of BE, EAC, and ESCC from normal tissue (AUC = 0.992), which achieved highly predictive ability in both internal (187 samples, AUC = 0.990) and external validation (184 samples, AUC = 0.978). The prognostic methylation classifier with 3 CpG and 2 CpG sites for EAC and ESCC respectively, could accurately estimate the prognosis of an individual patient and improved the predictive ability of the tumor node metastasis staging system. Overall, our study systematically analyzed large-scale methylation data and provided promising markers for the diagnosis and prognosis of esophageal cancer.

## INTRODUCTION

Esophageal cancer is the seventh most common cancer and the sixth leading cause of cancer death in the world [1]. Traditionally, esophageal cancer is subdivided into two histologic types, esophageal squamous cell carcinoma (ESCC) and esophageal adenocarcinoma (EAC), of which 88% of the cases are ESCC. The survival rate of both histologic types is extremely poor because of the late stage at diagnosis for most patients. The risk factors and molecular characteristics of ESCC and EAC are different [2]. The risk factors for ESCC include smoking and alcoholic beverages [3]. EAC is associated with obesity, gastric reflux and Barrett’s esophagus (BE) [4]. BE is a precursor lesion for EAC, where the squamous epithelium of the tubular esophagus is replaced by specialized intestinal-type columnar epithelium [5]. Genome sequencing studies have revealed that two histologic types of esophageal cancers exhibit distinct molecular profile at both genomic and epigenomic levels [6–8]. Genetic makers, such as somatic mutations, may be below the detection limit due to a low tumor load in early stages of cancer, and represent multiple cancer types and non-tumor conditions [9, 10]. Hence, genetic makers are thought to lack of specificity and sensitivity for a particular type of cancer. Epigenetic markers, especially DNA methylation, is thought as an ideal marker for early detection of cancer, as it has advantages of cancer-specific methylation patterns, occurrence in early cancer stages, biological stability, and technical repeatability [11]. However, type-specific epigenomic markers for diagnosis and prognosis of esophageal cancer have not been systematically compared and identified.

Aberrant methylation is common in various types of cancers, including esophageal cancer, which contributes to carcinogenesis [12]. Methylation-based markers have shown great potential for the diagnosis and survival prediction of solid tumors. In a previous study, a panel of DNA methylation markers differentiated tumor tissue and normal tissue in four common cancer types of breast, colon, liver, and lung, with an accuracy of more than 95% in two validation cohorts [13]. DNA hypermethylation in tumor suppressor genes have been observed in esophageal cancers including EAC and ESCC as well as in the EAC precursor lesion BE. Numerous methylation-based markers have been identified as potential biomarkers for diagnosis of BE and esophageal cancers, or predicting treatment response and prognosis of esophageal cancers [14, 15]. Remarkably, noninvasive methods based on nonendoscopic cell sampling devices have been used for seeking methylation markers for detecting BE and esophageal cancer [16, 17]. When a noninvasive device was applied to collect samples for detecting TFPI2 hypermethylation for BE diagnosis, the sensitivity and specificity were 82.2% and 95.7%, respectively [18]. A study found that VIM gene methylation is a highly sensitive biomarker for BE, which could be detected in esophageal brushings [19]. Moreover, using a novel swallowable balloon-based device that captures DNA samples for methylation analyses, a two marker panel of CCNA1 and VIM methylation for detecting BE and EAC from normal tissues provided more than 90% sensitivity and specificity [20]. However, previous study has suggested that ESCC has a stronger resemblance to head and neck squamous cell carcinoma (HNSC) than to EAC, and EAC more closely resembled to stomach adenocarcinoma (STAD) than ESCC [6]. Current studies focused on binary classification between tissues of BE and/or EAC, or ESCC with normal esophageal tissues. Although the samples from nonendoscopic devices may contain contaminations from nearby tissues, no previous studies have considered whether similar methylation patterns of the normal and cancerous tissues from adjacent organ may lead to misdiagnosis. Hence, tissue-specific methylation markers are absent and are needed to improve diagnosis.

In this present study, we aimed to identify diagnostic methylation markers for multiclass diagnosis of BE and two types of esophageal cancer from normal tissues, and prognostic methylation markers for survival prediction of esophageal cancer. Firstly, we identified tissue-specific methylation markers by removing the similar methylation patterns from the normal and cancerous tissues of adjacent organ. Then, we built a diagnostic methylation classifier for distinguishing these diseases. The diagnostic classifier was further validated in external datasets to assess the transportability and generalizability. Finally, we constructed prognostic methylation classifier for patients with esophageal cancer.

## RESULTS

### Diagnostic methylation classifier

The overall workflow and clinical characteristics of all patients is described in Supplementary Figure 1 and Supplementary Table 1. To identify tissue-specific methylation markers of normal squamous esophagus (NSE), BE, EAC, and ESCC, we included 564 samples of 4 tissue types of esophagus. To avoid the noise caused by the normal and cancerous tissues from adjacent organ, we also included 996 samples from HNSC and STAD. A total of 122,302 CpG sites was defined as tissue-specific markers for 4 tissue types of esophagus (Supplementary Figure 2). After feature selection by the least absolute shrinkage and selection operator (LASSO) model using 10 times random partitions and 10-fold cross-validation, we identified 458 CpG sites with different frequencies (Supplementary Figure 3). Twelve CpG sites with frequency greater than or equal to 9 were selected to construct the diagnostic methylation classifier (Table 1).

**Table 1:** Genomic information of 12 CpG sites for diagnostic methylation classifier.

**CpG**	**Gene symbol**	**Chromosome**	**Genomic coordinate**	**Relation to island**	**UCSC refgene group**
cg10078335	CAPN10	chr2	241535845	Island	Body
cg13257812	NA	chr3	27525884	Island	NA
cg04607372	NA	chr5	54523900	Island	NA
cg13441766	NA	chr5	134376442	Island	NA
cg13927501	TRIM31	chr6	30079090	OpenSea	Body
cg08858649	TRIM15	chr6	30139903	Island	Body
cg18080046	CLIC1	chr6	31704844	N_Shelf	TSS1500
cg14534279	NA	chr10	3329966	OpenSea	NA
cg06966660	TACC2	chr10	123923066	Island	Body
cg08436756	SHANK2	chr11	70781118	OpenSea	Body
cg01025720	ATP11A	chr13	113346439	S_Shore	Body
cg03474687	XRCC3	chr14	104179160	N_Shelf	5'UTR

**Figure 1:**
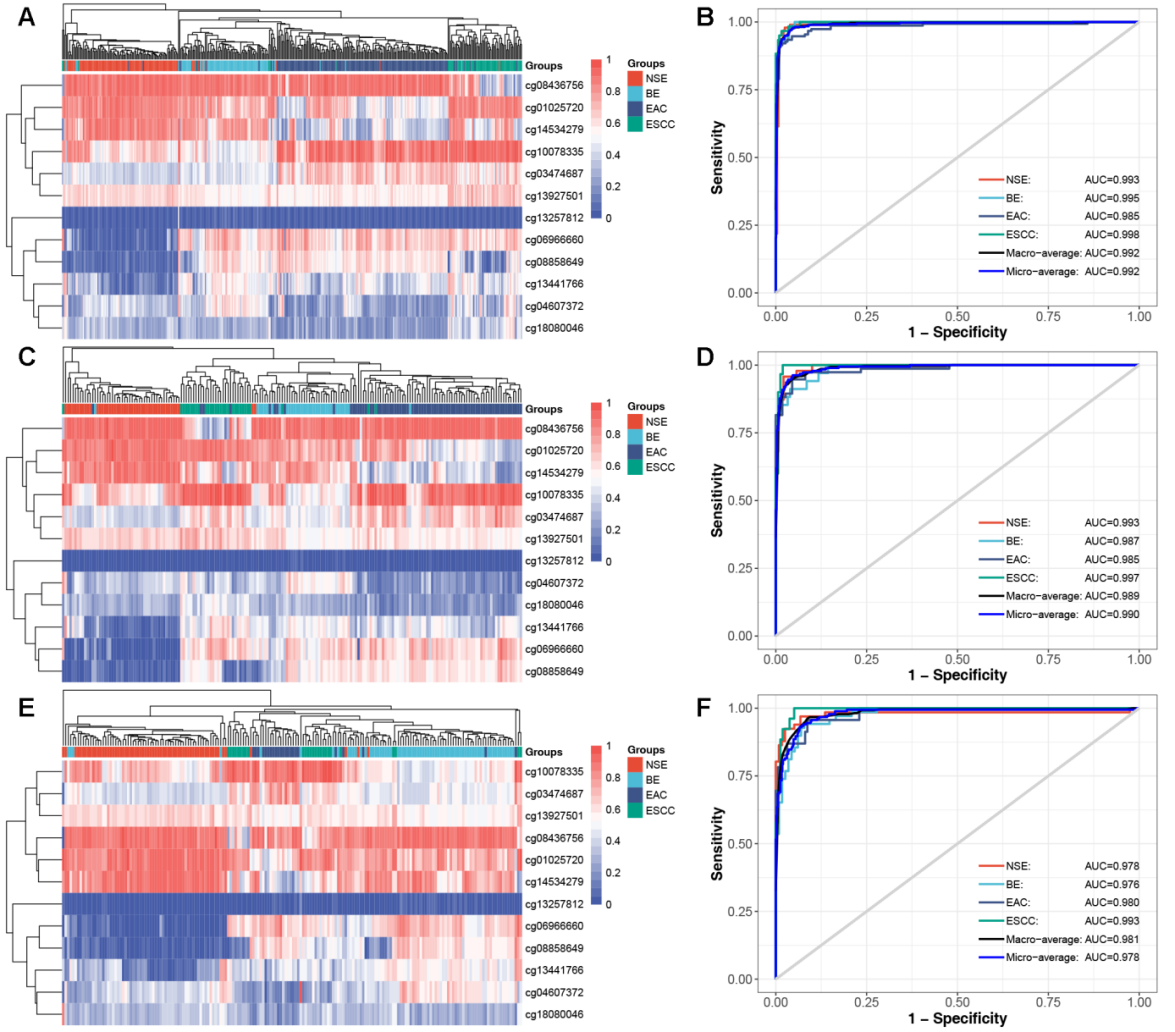
**Diagnostic methylation classifier can differentiate for NSE, BE, EAC, and ESCC.** Unsupervised hierarchical clustering and heatmap of 12 methylation markers selected for constructing diagnostic methylation classifier in (**A**) training (N=377), (**C**) test (N=187), and (**E**) validation set (N=184). ROC curve showing the high AUC in predicting four tissue types in (**B**) training, (**D**) test, and (**F**) validation set.

To evaluate the discriminative ability of 12 CpG-based diagnostic classifier, a multinomial logistic regression model (Supplementary Table 3) was built in training set (N = 377), which achieved total accuracy rate of 93.9% (95% confidence interval [CI]: 91.0%–96.1%, Table 2) and the micro-average Receiver Operating Characteristic (ROC) curve with an Area Under Curve (AUC) of 0.992 (Figure 1B). Then, the model derived from the training set was applied in test set (N = 187). The total accuracy rate was 93.1% (95% CI: 88.4%-96.3%, Table 3) and the micro-average AUC was 0.990 in the test set (Figure 1D). Next, we further evaluated the performance of the diagnostic classifier in validation set (N = 184). Consistently, the diagnostic classifier could effectively predict group membership in 159 (86.4%, 95% CI: 80.6%-91.0%) of 184 samples (Table 4), with a decreased but high AUC of 0.978 (Figure 1F). For 12 CpG sites, the distribution of methylated levels in the validation set was consistent with those in the training and test set (Supplementary Figure 4).

**Table 2:** Confusion matrix of training set.

**Hypothesized class**	**True class**				
	**NSE**	**BE**	**EAC**	**ESCC**	**Total**
NSE	**90**	2	3	1	96
BE	2	**64**	4	0	70
EAC	3	3	**143**	2	151
ESCC	1	0	2	**57**	60
Correct	90	64	143	57	354
Total	96	69	152	60	377
Accuracy rate (%)	93.75	92.75	94.08	95.00	93.90

**Table 3:** Confusion matrix of test set.

**Hypothesized class**	**True class**				
	**NSE**	**BE**	**EAC**	**ESCC**	**Total**
NSE	**45**	2	1	0	48
BE	1	**29**	2	0	32
EAC	1	3	**71**	1	76
ESCC	0	0	2	**29**	31
Correct	45	29	71	29	174
Total	47	34	76	30	187
Accuracy rate (%)	95.74	85.29	93.42	96.67	93.05

**Table 4:** Confusion matrix of validation set.

**Hypothesized class**	**True class**				
	**NSE**	**BE**	**EAC**	**ESCC**	**Total**
NSE	**60**	4	0	0	64
BE	4	**59**	1	1	65
EAC	1	6	**22**	7	36
ESCC	1	0	0	**18**	19
Correct	60	59	22	18	159
Total	66	69	23	26	184
Accuracy rate (%)	90.91	85.51	95.65	69.23	86.41

Overall, these results demonstrate that the diagnostic methylation classifier has a stable classification ability to predict the group membership of NSE, BE, EAC, and ESCC, and can eliminate the possible effect from normal and cancerous tissues of HNSC and STAD.

### Prognostic methylation classifier

**Figure 2:**
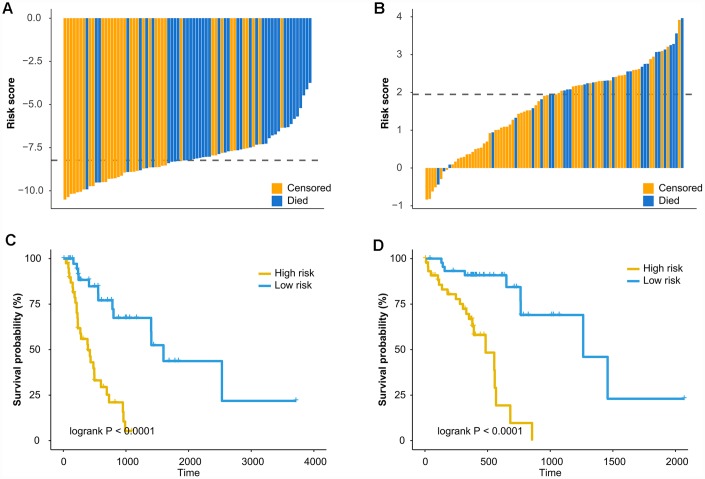
**Prognostic methylation classifier can predict overall survival of EAC and ESCC.** Waterfall plots show the risk scores of prognostic methylation classifier between high-risk and low risk patients for (**A**) EAC and (**B**) ESCC. The dash lines represent the median of the risk score. Kaplan-Meier curves were used of overall survival in high and low risk groups for (**C**) EAC and (**D**) ESCC. The cutoff values for the high and low risk groups were based on the median of the risk score.

The prognostic ability of methylation markers was determined for EAC (N = 79) and ESCC (N = 90). Firstly, a list of differential methylation CpG sites (DMCs) for EAC and ESCC was defined based on moderated t-statistics (|Δβ| > 0.2 and false discovery rate [FDR] < 0.05, Supplementary Figure 5A and 5B). Then, independent prognostic methylation markers were identified using multivariable Cox regression (Adjusted P < 0.05, Supplementary Figure 5C and 5D). Results that only one CpG site was overlapped between the independent prognostic markers of EAC (N = 3,980) and those of ESCC (N = 1,204), revealed that two types of esophageal cancer had distinct sets of prognostic methylation markers. Lasso-Cox model was utilized to select informative markers by resampling and cross-validation. Ultimately, prognostic methylation classifiers were constructed with 3 CpG sites for EAC and 2 CpG sites for ESCC (Table 5). The patients were classified into high-risk group and low-risk group based on the median of the risk score of classifiers (Figure 2A and Figure 2B). The Kaplan-Meier survival curve showed a significant difference in survival time between the two groups (Log-rank P < 0.0001, Figure 2C and Figure 2D). The 3 CpG-based and 2 CpG-based prognostic classifier for EAC (Hazard ratio [HR] = 5.164, Table 6) and ESCC (HR = 6.603, Table 7) respectively, were independent risk factors by multivariate Cox regression adjusting clinical risk factors. Time-dependent ROC curve analysis indicated that the predictive performance of the prognostic methylation classifiers was superior to those of clinical risk factors (Supplementary Table 4).

**Table 5:** Genomic information of CpG sites for prognostic methylation classifier.

**CpG**	**Gene symbol**	**Chromosome**	**Genomic coordinate**	**Relation to island**	**UCSC refgene group**
**EAC**					
cg01192745	NA	chr3	31239040	OpenSea	NA
cg19801256	ITGA1	chr5	52166469	OpenSea	Body
cg18276155	MCC	chr5	112504356	OpenSea	Body
**ESCC**					
cg14387626	NA	chr14	106331803	N_Shore	NA
cg04777726	PLEKHA4	chr19	49340489	Island	3'UTR

**Table 6:** Univariate and multivariate Cox regression analysis of the 3-CpG prognostic methylation classifier and clinical factors with overall survival of EAC.

**Risk factor**	**Univariate Cox**			**Multivariate Cox**	
	**HR (95% CI)**	**P value**		**HR (95% CI)**	**P value**
Age (> 60 vs ≤60)	0.986(0.962-1.009)	0.2283		0.986(0.960-1.014)	0.3275
Gender (male vs female)	0.847(0.299-2.400)	0.7553		0.552(0.158-1.928)	0.3520
BMI (> 25 vs ≤25)	1.019(0.983-1.058)	0.3056		1.063(1.015-1.112)	**0.0093**
Smoking (yes vs no)	1.133(0.606-2.117)	0.6953		1.317(0.681-2.547)	0.4138
Alcohol use (yes vs no)	0.516(0.281-0.948)	**0.0330**		0.616(0.304-1.245)	0.1771
Tumor stage (III/IV vs I/II)	2.238(1.151-4.351)	**0.0176**		2.028(0.930-4.420)	0.0753
Methylation classifier (high vs low risk)	5.661(2.639-12.145)	**< 0.0001**		5.164(2.199-12.130)	**0.0002**

**Table 7:** Univariate and multivariate Cox regression analysis of the 2-CpG prognostic methylation classifier and clinical factors with overall survival of ESCC.

**Risk factor**	**Univariate Cox**			**Multivariate Cox**	
	**HR (95% CI)**	**P value**		**HR (95% CI)**	**P value**
Age (> 60 vs ≤60)	1.763(0.826-3.765)	0.1428		1.536(0.681-3.463)	0.3011
Gender (male vs female)	10.290(1.358-78.001)	**0.0241**		3.508(0.407-30.207)	0.2533
BMI (> 25 vs ≤25)	0.727(0.283-1.868)	0.5082		1.211(0.453-3.240)	0.7031
Smoking (yes vs no)	2.113(0.939-4.754)	0.0706		1.257(0.526-3.003)	0.6066
Alcohol use (yes vs no)	2.169(0.750-6.277)	0.1530		4.562(1.311-15.880)	**0.0171**
Tumor stage (III/IV vs I/II)	2.987(1.432-6.230)	**0.0035**		1.980(0.873-4.492)	0.1020
Methylation classifier (high vs low risk)	7.354(2.962-18.257)	**< 0.0001**		6.603(2.407-18.116)	**0.0002**

**Figure 3:**
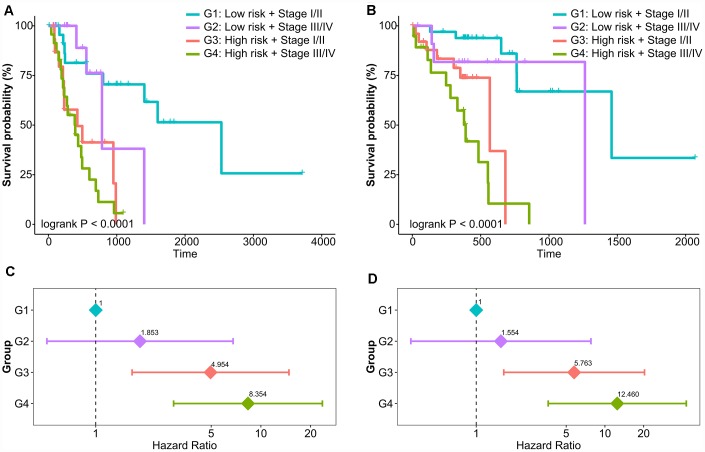
**Risk stratification combining prognostic methylation classifier and tumor stage in relation to overall survival of EAC and ESCC.** Kaplan-Meier curves of four risk levels for (**A**) EAC and (**B**) ESCC. Multivariate Cox model of four risk levels for (**C**) EAC and (**D**) ESCC adjusting for age, gender, BMI, smoking, and alcohol use.

Currently, tumor-node-metastasis (TNM) staging system remains the most valuable tool to predict prognosis for EAC and ESCC. Next, we assessed the association between our classifier and prognosis according to different TNM staging system. The results of the study showed that the high-risk group had poor prognosis in both early stage (stage I/II, Supplementary Figure 6A and 6B) and advanced stage (stage III/IV, Supplementary Figure 6C and 6D). In the risk stratification by combination of prognostic classifier and tumor stage, patients were divided into 4 risk levels of G1 (low-risk and early-stage), G2 (low-risk and advanced-stage), G3 (high-risk and early-stage), and G4 (high-risk and advanced-stage). Kaplan-Meier curves showed that patients in the different levels of risk stratification demonstrated significantly different prognoses (Log-rank P < 0.0001, Figure 3A and Figure 3B). A multivariable Cox model adjusted for clinical factors was built to determine whether the risk stratification was an independent prognostic factor, and groups of G3 and G4 were significantly different in overall survival compared with the reference group of G1 (Figure 3C and Figure 3D). In particular, patients had worse prognoses as risk levels increase (P for trend < 0.0001).

Overall, these results demonstrate that the prognostic methylation classifier can effectively predict the survival outcomes and improved risk stratification of patients with EAC and ESCC.

## DISCUSSION

In the present study, we systematically analyzed genome-wide methylation data from 1,744 samples to identify and validate specific diagnostic methylation markers for BE, EAC, and ESCC by eliminating contamination from the adjacent organ of head and neck and stomach. A panel of DNA methylation markers, selected by the LASSO method, achieved a highly predictive ability for distinguishing BE and EAC and ESCC from normal tissues in both internal and external validations. Prognostic methylation classifier for EAC and ESCC specifically was developed to classify the patients into high risk and low risk, which could accurately estimate the prognosis of an individual patient

Methylation-based markers researches for esophageal cancer have mainly been focused on hypermethylation in promoter region CpG island of numerous tumor suppressor genes, such as APC and CDKN2A, which thus were thought to be potential biomarkers for the diagnosis of BE and esophageal cancer [14, 15]. However, these methylation-based markers were not specific and sensitive as hypermethylation also occur frequently in other cancer types. Other studies have evaluated the utility of genome-wide methylation data to discovery methylation-based markers for esophageal cancer. A study examined the methylation status of 27,578 CpG sites in 94 normal esophageal, 77 BE and 117 EAC tissue samples [21]. Results suggested that the AUCs for discriminating BE and EAC from normal esophageal tissue were 0.965 and 0.973, respectively, but the difference between the BE and EAC tissues was less clear. A study with 112 samples of HumanMethylation450 data identified five hypermethylated CpG sites as candidate biomarkers for ESCC (AUC = 0.85), which were further validated in 94 pairs tumor and adjacent normal tissues using the targeted bisulfite sequencing method [22]. However, most of these studies only focus on binary classifications and compared a single disease of BE or EAC or ESCC, or a combination of BE and EAC, to normal esophageal tissue. Moreover, considering the limitations in the numbers and types of samples included in these studies, there is need for further study on novel methylation-based markers for BE and esophageal cancer. In this study, we analyzed a large scale of genome-wide methylation data to identify tissue-specific methylation markers of BE and two types of esophageal cancer. The diagnostic methylation classifier constructed by the LASSO method, achieved high accuracy in the internal and external validation, and had a better performance compared with previous markers. Our diagnostic methylation classifier is the only one for multinomial classification that can effectively distinguish BE and two types of esophageal cancer from normal tissues.

Endoscopy is the gold standard for the detection and diagnosis of BE and esophageal cancer but is not a cost-effective or feasible and noninvasive screening method. Given the need of cost-benefit, nonendoscopic cell-collecting devices have been developed by capturing samples from the esophagus to identify molecular biomarkers. One of such devices is named *Cytosponge*, which is widely used to sample cells from the esophagus [23]. The captured samples are analyzed for molecular markers that show a diagnostic accuracy comparable to endoscopy. This procedure suggests that the samples from such devices may contain contaminations from nearby tissues when the sampling device is withdrawn from the stomach to the mouth. In this regard, our study identified sets of tissue-specific methylation markers for BE and esophageal cancer by removing the similar methylation patterns from the normal esophageal tissues and adjacent cancer types of HNSC and STAD. Our diagnostic methylation classifier has clinical applicability for screening BE and esophageal cancer by these noninvasive devices, which required tissue-specific and effective markers. Furthermore, as our diagnostic methylation classifier used a small number of markers with precise genomic position, further studies could detect methylation level of these markers in clinical samples using the cheaper technique such as targeted bisulfite sequencing rather than expensive microarrays.

Among our methylation signatures, some genes have been reported in the onset and progression of BE and esophageal cancer or the prognosis of esophageal cancer, such as TRIM15, TACC3, SHANK2, and MCC [24–28]. For example, one of the diagnostic CpG sites (cg08858649) was within CpG island of TRIM15 gene, and single gene methylation had a *c*-statistic of 0.91 (95% CI: 0.88-0.99) in discriminating the combination of EAC and BE from normal mucosa [24]. A prognostic methylation marker (cg18276155) for EAC was located at a classic tumor suppressor gene of esophageal cancer, MCC gene. Several reports have described high rates of loss of heterozygosity at MCC in esophageal cancer [27, 28]. Although the link between aberrant methylation and gene alterations is not yet known, our study suggested that aberrant methylation of MCC gene might contribute to progression of EAC via epigenetic regulation. Further mechanism studies are warranted to offer a better understanding of the biological roles of these CpG sites on the molecular pathogenesis, and ultimately improve the diagnosis and prognosis of esophageal cancer.

To the best of our knowledge, this is the first attempt to build a diagnostic classifier with a high predictive ability to differentiate EAC and ESCC as well as EAC precursor lesion BE from normal tissues and adjacent cancer types of HNSC and STAD. Our study also has some limitations. First, the discriminative ability of diagnostic methylation classifier in external validation set was slightly decreased compared to those in internal validation. Meanwhile, prognostic methylation classifier was constructed based on a small sample size, and not verified in external datasets because of the limited available data. Further studies with more samples are needed to enhance the statistical power and predictive accuracy. Second, the mechanistic contributions of some methylation signatures to the development and progression of esophageal cancer remain unknown, further validation efforts on their biological functions may provide novel pathogenic mechanisms and therapeutic targets.

In summary, panels of methylation markers have the potential for diagnosis and prognosis of Barrett’s esophagus and esophageal cancer. Although substantial studies are still required to verify potential values of these methylation markers in noninvasive detection before this can be implemented into clinical practice, our study provided a methodology of choice for constructing diagnostic and prognostic methylation classifier for esophageal cancer.

## MATERIALS AND METHODS

### Data source

DNA methylation data from HumanMethylation450 were obtained from The Cancer Genome Atlas (TCGA, https://cancergenome.nih.gov/) and the Gene Expression Omnibus (GEO, https://www.ncbi.nlm.nih.gov/geo/) datasets. The level 3 DNA methylation data from three TCGA projects of ESCA, HNSC, and STAD were downloaded from the legacy archive of the Genomic Data Commons (GDC, https://portal.gdc.cancer.gov/). Six datasets were downloaded from GEO datasets, with the GEO accession numbers GSE52826, GSE72874, GSE74693, GSE79366, GSE81334, and GSE104707 [29–35]. Three TCGA projects, GSE72874, and GSE104707 were used for identifying tissue-specific methylation markers and constructing diagnostic methylation classifier, including 143 NSE, 103 BE, 228 EAC, 90 ESCC, 528 HNSC, 50 normal tissues of HNSC, 395 STAD, and 23 normal tissues of STAD. External validation sets of GSE52826, GSE74693, GSE79366, and GSE81334 were used to validate the predictive performance of the diagnostic classifier, including 66 NSE, 69 BE, 23 EAC, and 26 ESCC. The methylation levels of each CpG site was represented by beta-value, which was the ratio of the methylated probe intensity and the overall intensity (the sum of the methylated and unmethylated probe intensities). The CpG sites that were from the X and Y chromosomes, or were known to have common SNPs, or were cross-hybridized with multiple genomic loci, were removed.

### Diagnostic methylation classifier

Firstly, we identified to a list of tissue-specific markers for NSE, BE, EAC, ESCC, HNSC, normal tissues of HNSC, STAD, and normal tissues of STAD in discovery dataset. Differential methylation analysis of each CpG site was tested by pairwise-comparisons using moderated t-statistics. For each pairwise-comparison, the differentially methylated CpG sites were defined as those having FDR with the Benjamini-Hochberg procedure of less than 0.05. The CpG sites, that in a specific tissue type were significantly different in all the comparisons with the other 7 tissue types, were defined as tissue-specific markers. The tissue-specific markers and samples of NSE BE, EAC, and ESCC were retained for the subsequent analysis. The LASSO was applied to select the panel of tissue-specific markers for constructing diagnostic classifier. The full dataset was randomly partitioned into training and test sets, at a 2:1 ratio, and the procedures for the random partitions were repeated 10 times to generate 10 different training-test sets to minimize the random error. In each training set, a multinomial logistic regression was built using a grouped lasso penalty, with the estimated tuning parameters. The optimal tuning parameters were evaluated using a 10-fold cross-validation in the training set, and a lambda with an accuracy that was one standard error below the maximum accuracy was adopted. Markers with a frequency greater than or equal to 9 were chosen to build the diagnostic classifier for multiclassification. Then, a multinomial logistic model was built in training set and evaluated in the test set. The built model was further applied to the validation set to verify the transportability and generalizability of diagnostic methylation classifier. A confusion matrix and a ROC using one-vs-all approach were generated in all datasets. The performance of the diagnostic classifier was evaluated by AUC.

### Prognostic methylation classifier

Prognostic prediction was performed for EAC and ESCC. First, we used the moderated t-statistics to identify the DMCs between cancer and normal samples, with an absolute value of differential methylated levels (|Δβ|) greater than 0.2 and an FDR < 0.05. Among these DMCs, independent prognostic methylation markers (P < 0.05) were defined using Cox proportional hazards model by adjusting for age, gender, BMI, smoking, alcohol use, and American Joint Commission on Cancer (AJCC) tumor stage. Then, we adopted LASSO-Cox models by repeating 100 times of subsampling 75% of the patients without replacement and 5-fold cross-validation to select prognostic markers. The selected markers with frequency more than 30 were used to construct the prognostic classifier, and the patients were categorized into high and low groups based on the median risk score of the prognostic classifier. The Kaplan-Meier log-rank test, multivariable Cox model and time-dependent ROC analysis were performed to evaluate the predictive ability of prognostic methylation classifier.

### Statistical analysis

All the statistical tests were two-sided, and a P value < 0.05 was considered statistically significant unless otherwise specified. All the analyses were implemented in R version 3.5.1. The R packages used in the analyses are listed in Supplementary Table 2.

## Supplementary Material

Supplementary Figures

Supplementary Tables
