# Supplementary material for: Specific DNA methylation markers in the diagnosis and prognosis of esophageal cancer
Source: Aging (Albany NY). 2019 Dec 13;11(23):11640–58. doi: 10.18632/aging.102569 (PMC6932928; doi:10.18632/aging.102569)
Supplement: Supplementary Figures [file aging-11-102569-s002..pdf]

SUPPLEMENTARY FIGURES

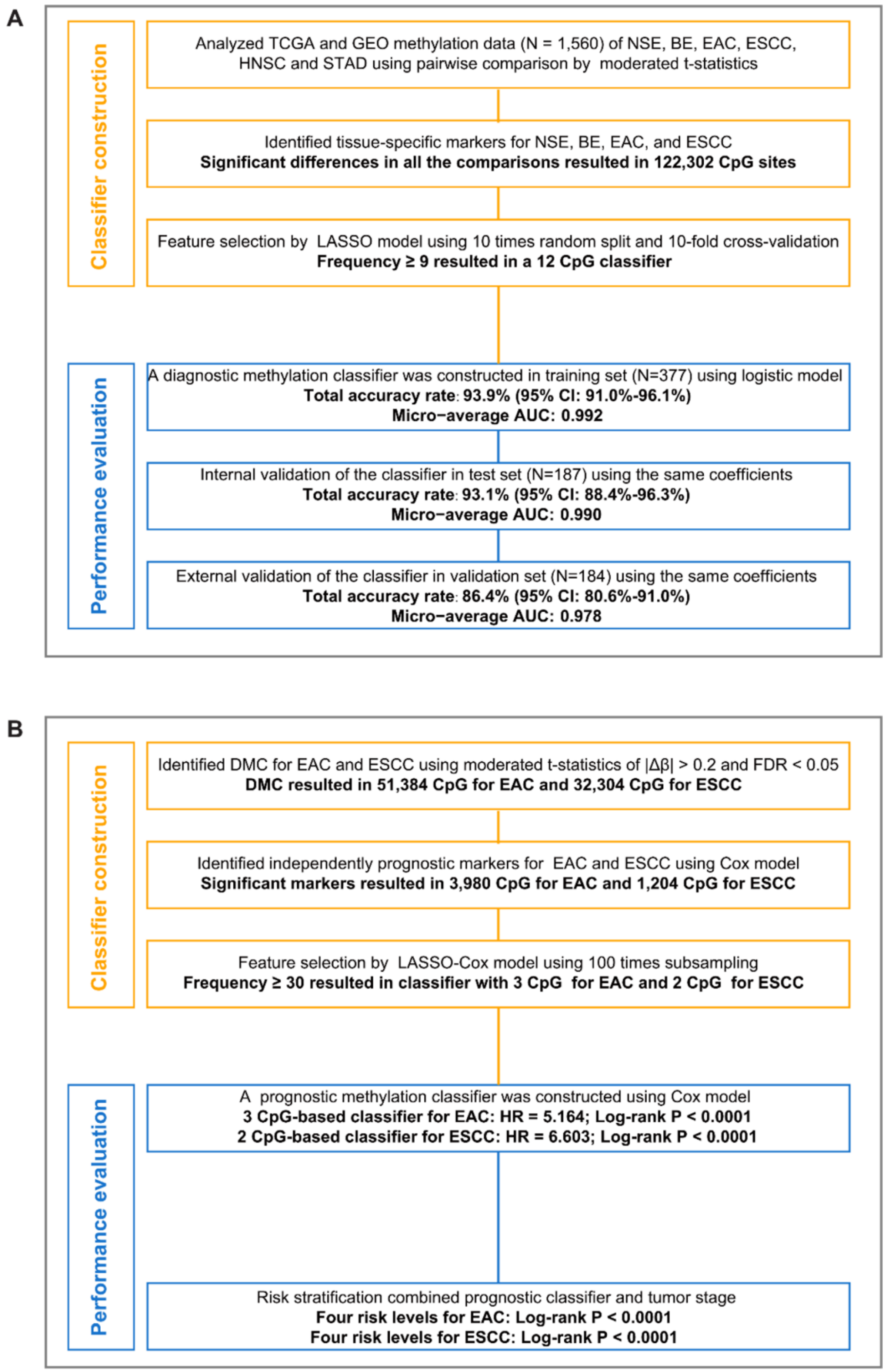

Supplementary Figure 1. Overall workflow of the various analyses performed in this study. Construction of (**A**) diagnostic methylation classifier and (**B**) prognostic methylation classifier.

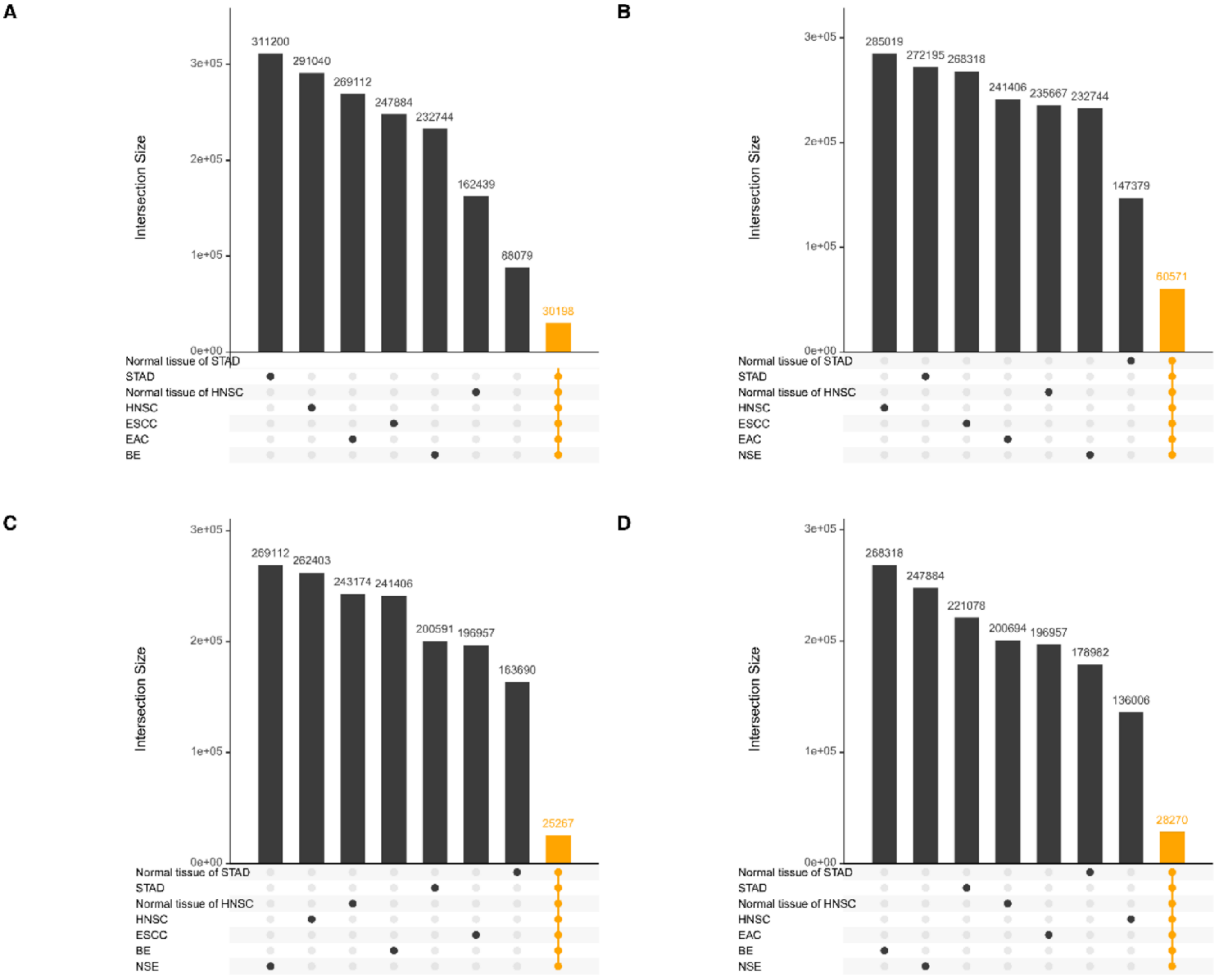

Supplementary Figure 2. Statistics of tissue-specific methylation markers for four tissue types of esophagus. Numbers of tissue-specific methylation markers were identified by moderated t-statistics for group of (**A**) NSE, (**B**) BE, (**C**) EAC, and (**D**) ESCC. Tissue-specific markers were defined as overlapping CpG sites (*orange bar*) that were significantly differential methylated (FDR < 0.05) in all the pair-wise comparisons (*black bar*) with the other seven tissue types.

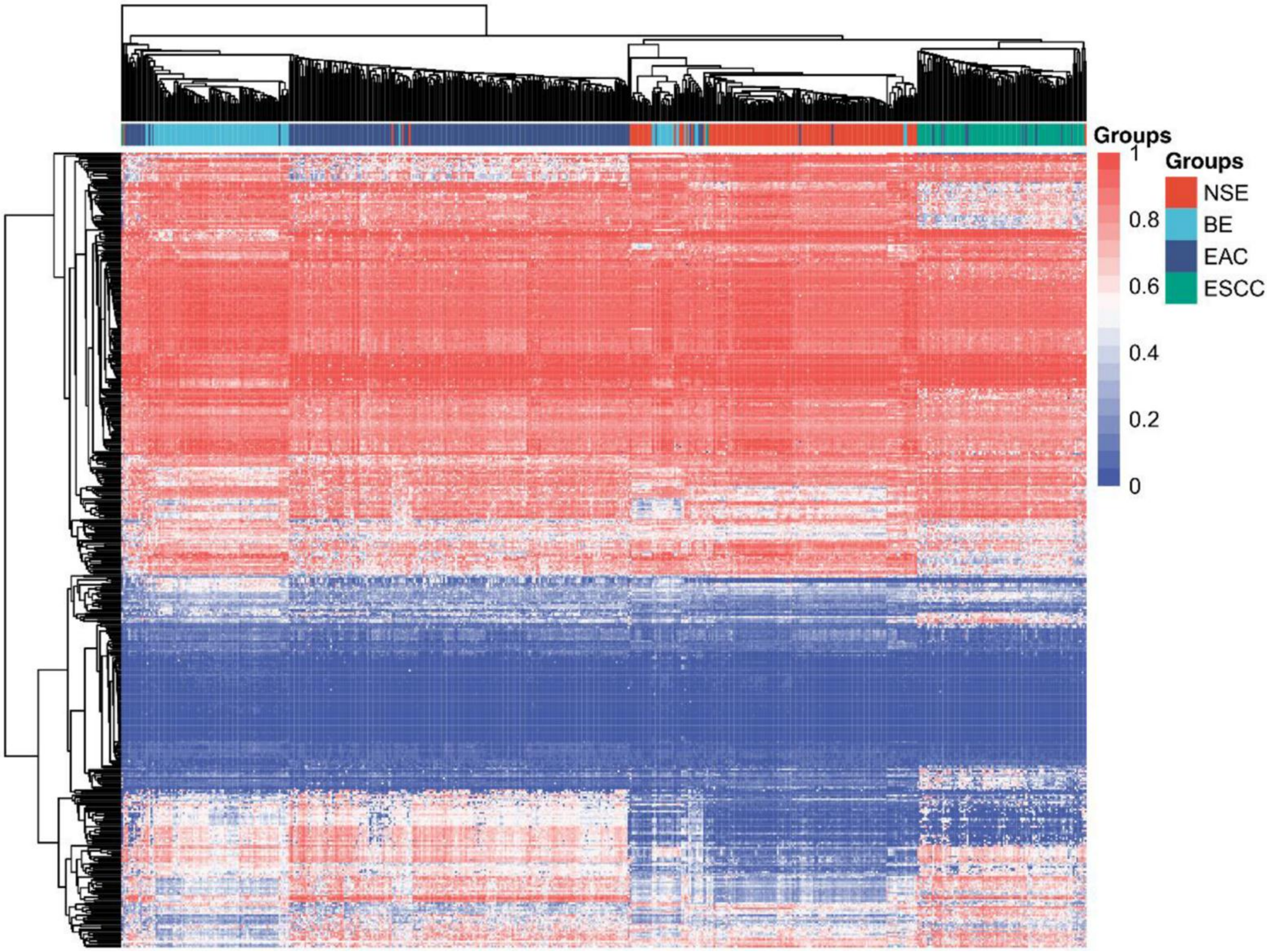

Supplementary Figure 3. The heatmap showing the methylation levels of 458 diagnostic CpG sites selected by LASSO in training and test set across four tissue types of esophagus. Row represents specific markers (N = 458). Column represents four types of samples (N = 564).

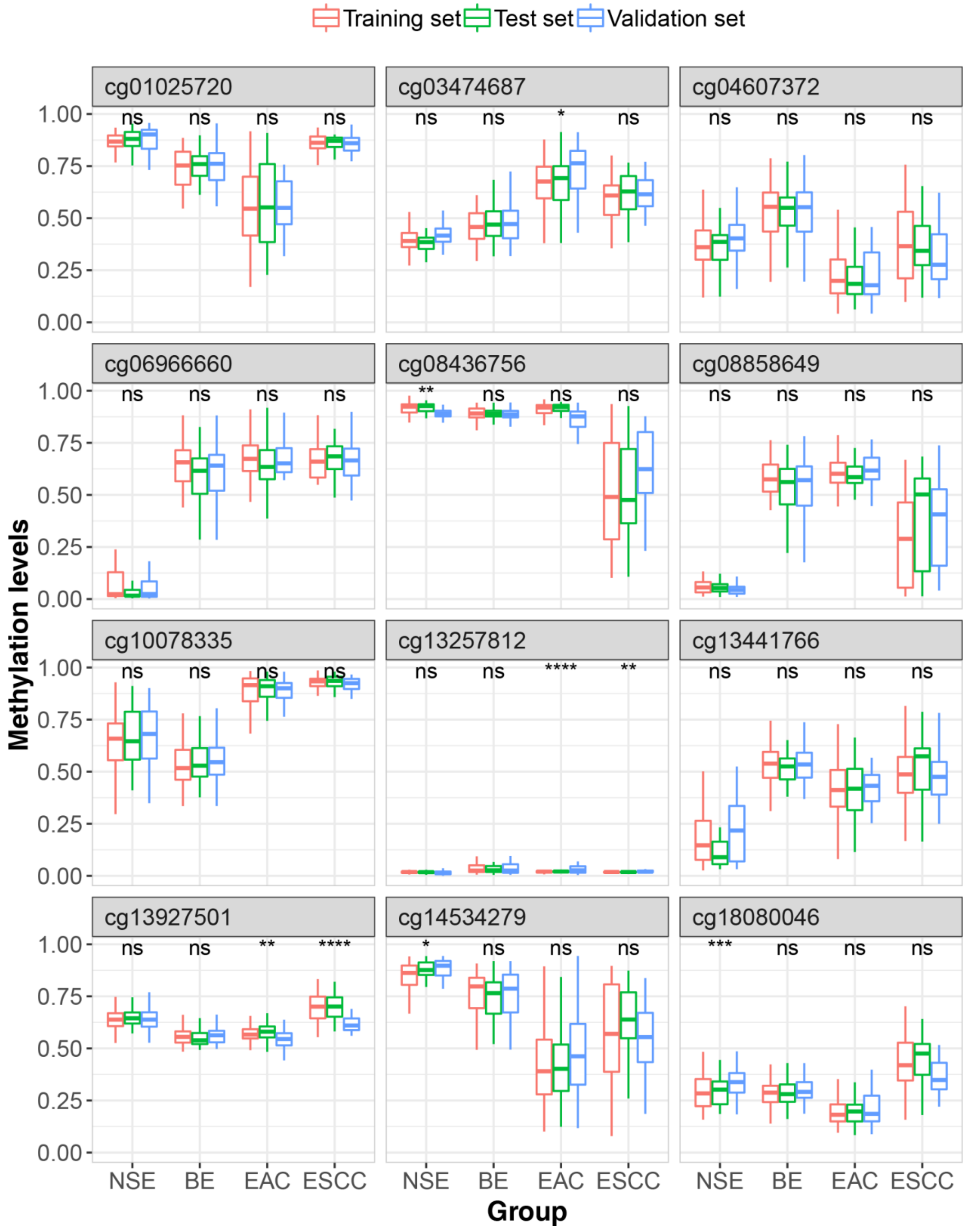

Supplementary Figure 4. Distribution of methylation levels of 12 diagnostic CpG sites across four tissue types of esophagus in training, test, and validation set. Symbols indicate statistical significance of one-way analysis of variance: ns, p > 0.05; *, p ≤ 0.05; **, p ≤ 0.01; ***, p ≤ 0.001; ****, p ≤ 0.0001.

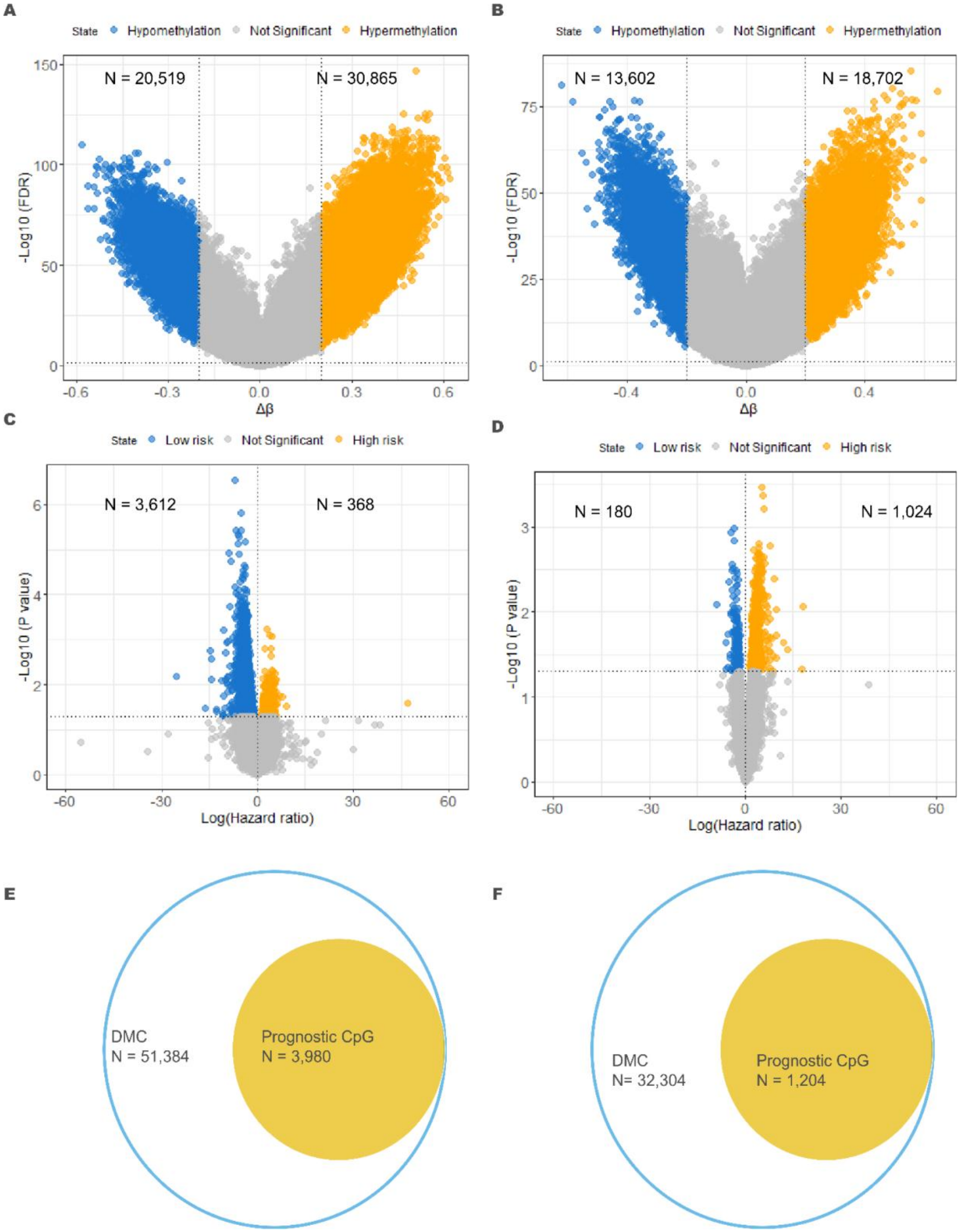

Supplementary Figure 5. Identification of prognostic methylation markers for EAC and ESCC. Different methylated CpG (DMC) sites in tumor and normal samples by moderated t-statistics ($|\Delta\beta| > 0.2$ and FDR <0.05) for (**A**) EAC and (**B**) ESCC. Independently prognostic CpG sites by multivariable Cox regression (P < 0.05) for (**C**) EAC and (**D**) ESCC. Numbers of prognostic CpG sites in DMC for (**E**) EAC and (**F**) ESCC.

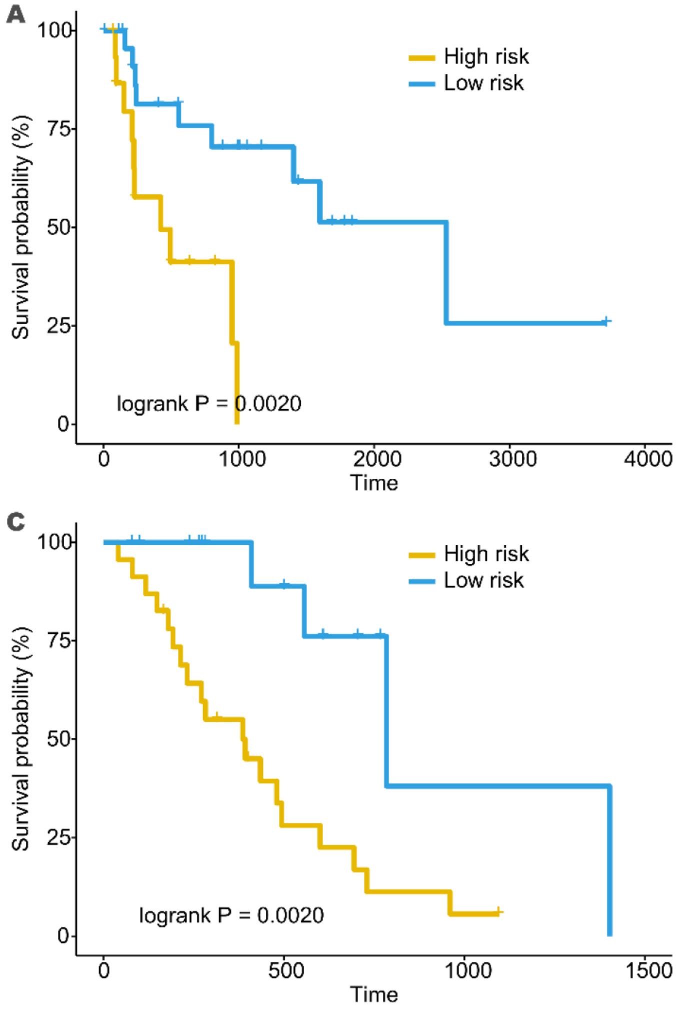

Supplementary Figure 6. Prognostic methylation classifier and overall survival in early stage and advanced stage. Overall survival curves of (**A**) EAC patients and (**B**) ESCC patients in early stage. Overall survival curves of (**C**) EAC patients and (**D**) ESCC patients in advanced stage.
