# Supplementary material for: Specific DNA methylation markers in the diagnosis and prognosis of esophageal cancer
Source: Aging (Albany NY). 2019 Dec 13;11(23):11640–58. doi: 10.18632/aging.102569 (PMC6932928; doi:10.18632/aging.102569)
Supplement: Supplementary Tables [file aging-11-102569-s001..pdf]

SUPPLEMENTARY TABLES

Supplementary Table 1. Clinical characteristics of included samples (N = 1,744).

Characteristic	Esophagus				HNSC		STAD	
	NSE	BE	EAC	ESCC	Normal	Tumor	Normal	Tumor
Total (n)	209	172	251	116	50	528	23	395
Age (Mean ± SD)	63.0 ± 13.3	64.3 ± 12.8	65.0 ± 11.3	59.8 ± 10.5	62.6 ± 10.7	61.4 ± 11.9	64.3 ± 11.7	65.7 ± 10.7
Gender -NO. (%)								
Female	39 (18.7%)	30 (17.4%)	24 (9.6%)	14 (12.1%)	12 (24.0%)	142 (26.9%)	4 (17.4%)	136 (34.4%)
Male	163 (78.0%)	138 (80.2%)	222 (88.4%)	90 (77.6%)	38 (76.0%)	386 (73.1%)	19 (82.6%)	259 (65.6%)
Missing data	7 (3.3%)	4 (2.3%)	5 (2.0%)	12 (10.3%)	0 (0.0%)	0 (0.0%)	0 (0.0%)	0 (0.0%)
Smoking - NO. (%)								
No	29 (13.9%)	4 (2.3%)	76 (30.3%)	39 (33.6%)	40 (80.0%)	230 (43.6%)	8(34.8%)	0 (0.0%)
Yes	55 (26.3%)	11 (6.4%)	118 (47.0%)	51 (44.0%)	10 (20.0%)	298 (56.4%)	13 (56.5%)	0 (0.0%)
Missing data	125 (59.8%)	157 (91.3%)	57 (22.7%)	26 (22.4%)	0 (0.0%)	0 (0.0%)	2 (8.7%)	395 (100.0%)
Alcohol use - NO. (%)								
No	15 (7.2%)	1 (0.6%)	55 (21.9%)	24 (20.7%)	13 (26.0%)	165 (31.2%)	9 (39.1%)	0 (0.0%)
Yes	51 (24.4%)	14 (8.1%)	111 (44.2%)	64 (55.2%)	36 (72.0%)	352 (66.7%)	12 (52.2%)	0 (0.0%)
Missing data	143 (68.4%)	157 (91.3%)	85 (33.9%)	28 (24.1%)	1 (2.0%)	11 (2.1%)	2 (8.7%)	395 (100.0%)
AJCC stage -NO. (%)								
I	-	-	8 (3.2%)	6 (5.2%)	-	27 (5.1%)	-	52 (13.2%)
II	-	-	21 (8.4%)	56 (48.3%)	-	74 (14.0%)	-	125 (31.6%)
III	-	-	26 (10.4%)	29 (25.0%)	-	82 (15.5%)	-	174 (44.1%)
IV	-	-	5 (2.0%)	7 (6.0%)	-	270 (51.1%)	-	33 (8.4%)
Missing data	-	-	191 (76.1%)	18 (15.5%)	-	75 (14.2%)	-	11 (2.8%)

Supplementary Table 2. R packages used in various analyses.

R packages	Function in analyses
minfi	Data pre-processing
limma	Moderated t-statistics
IlluminaHumanMethylation450kanno.ilmn12.hg19	Annotation of CpG sites
pheatmap	Hierarchal clustering and heatmap
glmnet	LASSO
nnet	Multinomial logistic model
multiROC	ROC curves across multi-class classifications
survival	Cox model
timeROC	Time-dependent ROC analysis

Supplementary Table 3. Coefficients of multinomial logistic model derived from training set.

	BE	EAC	ESCC
(Intercept)	-17.53	-20.18	-33.93
cg06966660	2.99	0.41	6.80
cg08436756	-1.40	-5.09	-13.01
cg08858649	5.02	13.08	6.05
cg10078335	-4.93	6.01	7.78
cg13257812	35.65	-25.59	-0.86
cg01025720	0.32	-3.91	0.21
cg03474687	17.26	17.39	10.93
cg04607372	3.64	-2.61	-3.08
cg13441766	11.80	8.66	6.92
cg13927501	4.39	20.38	42.16
cg14534279	3.32	-0.74	0.63
cg18080046	-4.07	-13.41	-2.01

Supplementary Table 4. Estimation of time-dependent AUC of clinical factors and prognostic methylation classifier for EAC and ESCC.

Risk factor	EAC		ESCC	
	3 year-AUC (%)	SE	3 year-AUC (%)	SE
Age	43.73	11.52	74.80	9.62
Gender	50.52	5.24	82.34	13.72
BMI	54.26	9.51	1.04	1.16
Smoking	54.41	8.31	53.09	14.55
Alcohol use	42.04	8.40	39.80	5.27
Tumor stage	75.14	8.32	69.55	16.08
Methylation classifier	93.82	3.24	97.47	3.18
